# Detection of Simple and Pattern Regularity Violations Occurs at Different Levels of the Auditory Hierarchy

**DOI:** 10.1371/journal.pone.0043604

**Published:** 2012-08-20

**Authors:** Miriam Cornella, Sumie Leung, Sabine Grimm, Carles Escera

**Affiliations:** 1 Institute for Brain, Cognition and Behavior (IR3C), University of Barcelona, Catalonia, Spain; 2 Cognitive Neuroscience Research Group, Department of Psychiatry and Clinical Psychobiology, University of Barcelona, Catalonia, Spain; Neuroscience Campus Amsterdam, VU University, The Netherlands

## Abstract

Auditory deviance detection in humans is indexed by the mismatch negativity (MMN), a component of the auditory evoked potential (AEP) of the electroencephalogram (EEG) occurring at a latency of 100–250 ms after stimulus onset. However, by using classic oddball paradigms, differential responses to regularity violations of simple auditory features have been found at the level of the middle latency response (MLR) of the AEP occurring within the first 50 ms after stimulus (deviation) onset. These findings suggest the existence of fast deviance detection mechanisms for simple feature changes, but it is not clear whether deviance detection among more complex acoustic regularities could be observed at such early latencies. To test this, we examined the pre-attentive processing of rare stimulus repetitions in a sequence of tones alternating in frequency in both long and middle latency ranges. Additionally, we introduced occasional changes in the interaural time difference (ITD), so that a simple-feature regularity could be examined in the same paradigm. MMN was obtained for both repetition and ITD deviants, occurring at 150 ms and 100 ms after stimulus onset respectively. At the level of the MLR, a difference was observed between standards and ITD deviants at the Na component (20–30 ms after stimulus onset), for 800 Hz tones, but not for repetition deviants. These findings suggest that detection mechanisms for deviants to simple regularities, but not to more complex regularities, are already activated in the MLR range, supporting the view that the auditory deviance detection system is organized in a hierarchical manner.

## Introduction

In order to give optimal responses to changes in the environment, auditory inputs need to be processed in a fast and efficient way. In humans, automatic auditory deviance detection has traditionally been associated with the mismatch negativity [1], a component of the auditory evoked potential (AEP) occurring 100–250 ms after stimulus onset [2]. MMN is generated by sources located in the supratemporal plane [3]–[4] and the prefrontal cortex [5]. However, automatic auditory deviance detection mechanisms exist at different anatomical levels and temporal scales [6]–[7]. For example, single-unit recordings in primary auditory cortex (A1) neurons of the cat exhibit a property termed “stimulus-specific adaptation” (SSA), that is, their spiking rate decreases when a stimulus is repeated, but increases again when a different stimulus is presented [8]. Such an increase is not due to a mere release from refractoriness, but it is considered the result of a genuine deviance detection process [9]–[10]. Further studies have identified SSA in subcortical structures, such as the medial geniculate body of the thalamus [11]–[12] and the inferior colliculus [13]–[15].

In humans, a better correlate to the SSA responses observed in animals might lie in the components of the middle latency response (MLR) of the AEP instead of in the MMN. First, SSA responses observed in animals are not related to the NMDA receptor function, which has been linked to MMN [16]–[17]. Second, MLR responses occur 10–50 ms after stimulus onset [2], and are thought to originate in A1 or secondary areas of the auditory cortex [18]. By using the classic oddball paradigm, modulations in the MLR components have been observed depending on the acoustic feature that violated the regularity. A deviance-related enhancement has been reported at the Na component for location [19]–[20], at Pa for band-pass filtered noise bursts [21], at Nb for frequency [22], [23], and at the Na-Pa complex for intensity [24]. Therefore, it is conceivable that, at least for simple features, deviance detection processes might be activated at early stages, supporting the view of a hierarchical novelty detection system [22], [25].

While a modulation of the MLR has been observed to simple regularity violations in oddball paradigms, it is yet to be determined whether deviations to complex regularities might also be detected at these earlier stages. To answer this question, we examined different levels of auditory change detection in a tone-alternation paradigm [26]. We used sequences of sounds that were alternating in frequency, but contained rare violations in the form of repetitions. Previous studies have shown that MMN is elicited when a repetition breaks the tone-alternating regularity [26]–[29]. In addition to the tone repetitions, we introduced occasional changes in the interaural time difference (ITD) of the tones, leading to a perceived sound location change. This way we could examine a regularity based on a feature representation in which an enhancement of the Na component to the deviants was already reported [19], [20]. Therefore the tone-alternating sequence contained two types of regularities, a “simple” regularity accounting for the perceived location of the stimuli, and a “pattern” regularity represented by the alternating tones. Deviance detection was studied at the level of the MLR and the MMN for both types of regularity violations.

## Materials and Methods

### Ethics Statement

All participants gave written informed consent. The experiment was approved by the Ethical Committee of the University of Barcelona, and was in accordance with the Code of Ethics of the World Medical Association (Declaration of Helsinki).

### Participants

Data were collected from twenty-five healthy participants (mean age: 25.6; range: 20–33 years; 6 males), who participated in the experiment for payment (6€ per hour). All participants were tested for normal hearing and had a mean hearing threshold below 25 dB SPL in the audiometry (between 400 and 3000 Hz). Additionally, they were asked to complete a health questionnaire to screen for any history of neurological or psychiatric disease. One participant was excluded due to a poor MLR signal, so that the final number of subjects used for analysis was 24.

### Stimuli and Procedure

**Figure 1:**
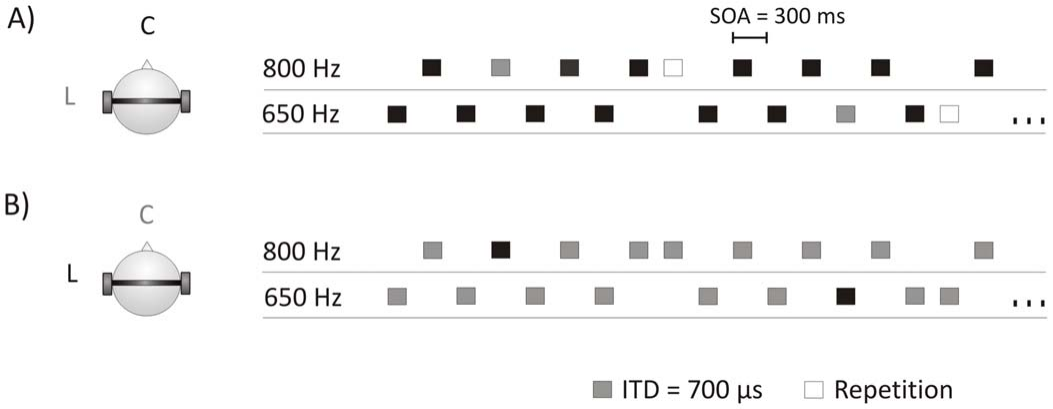
Experimental design. A) Tone-alternation sequence. B) Reverse block. Letters *C* (center) and *L (*left) indicate the perceived location of the sound for standards (in black), and deviants (in grey).

The stimulus sequence consisted of two alternating pure tones of 650 and 800 Hz. Both tones had a duration of 50 ms (5 ms rise, 10 ms fall times). Tones were delivered binaurally through headphones (Beyerdynamic DT48A; Beyerdynamic) at 70 dB SPL, with a constant stimulus-onset asynchrony (SOA) of 300 ms. Repetition deviants consisted of a repetition of either one of the two tones, and occurred randomly within the sequence with a probability of 0.05 (0.025 for each frequency). Within the same sequence, feature deviants were presented by introducing an ITD of 700 µs delay on the right channel for each tone, so that the sound source location was perceived as coming from the left. ITD deviants also occurred with a probability of 0.05 (0.025 for each frequency). To control for physical stimulus properties, a reverse block was introduced. This block consisted of the same tone-alternation sequence with occasional repetitions, but with the ITD changes applied to the standard tones. The deviants in this reverse block occurred with a probability of 0.05 and had an ITD of 0 µs. This way, the standard tones in the reverse block had the same physical properties as the deviants in the tone-alternation blocks, so that the only difference between them was their role within the sequence (Figure 1). Stimulus presentation was controlled via MATLAB, using the Psychophysics Toolbox extensions [30]–[32].

During recording participants sat comfortably in an electrically shielded and sound-attenuated room. Participants were asked to ignore the sound stimuli while watching a silent movie with subtitles. The tone-alternating sequences were presented in 10 blocks, each with 2280 stimuli. The total number of deviants was 2280, that is, 1140 for the simple regularity and 1140 for the pattern regularity violations. In the reverse block 1200 stimuli were presented, which included 1140 standard tones. The reverse block was introduced either in the first half of the experiment or in the second half. In all blocks, deviants appeared pseudo-randomly, so that there were at least 3 standard tones at the beginning of the sequence and in between deviants. During the experiment, small breaks (circa 5 minutes) were introduced every two blocks.

### Data Acquisition

Electroencephalographic (EEG) recordings were obtained with Neuroscan 4.4 acquisition software from 62 scalp electrodes mounted on an elastic nylon cap (Quik-Cap, Compumedics Neuroscan) according to the 10–20 system. Additionally, two electrodes were placed on left and right mastoids (M1 and M2). The electrooculogram (EOG) was measured with two bipolar electrodes placed above and below the left eye (VEOG), and two horizontal electrodes placed on the outer canthi of the eyes (HEOG). An electrode placed on the tip of the nose served as online reference. All electrode impedances were kept below 10 kΩ. EEG signals were amplified using a SynAmpsRT amplifier (NeuroScan, Compumedics, Charlotte, NC) with an online bandpass filter from 0.05 to 500 Hz, and were digitized with a sampling rate of 2000 Hz.

### Analysis

For the MMN analysis, data were filtered using a band-pass FIR filter from 1 to 30 Hz, and were offline re-referenced to the linked mastoids. Epochs of 400 ms were used, which included a −100 ms baseline relative to stimulus onset. Epochs with absolute amplitudes larger than ±80 µV at any electrode and any point in time were rejected from further analysis. Epochs were averaged separately for standard and deviant tones. For the pattern regularity violation, we compared the responses to rare tone repetitions with those to the immediately preceding standard tones. For the simple regularity violation, ITD deviants were compared to the standard tones of the reverse block. This way, for both types of regularity violations, deviants were compared to standards that had the same physical characteristics. A 40 ms window around the grand average peak latency was used to calculate individual mean amplitudes elicited by each subject at the electrode Fz. For the simple regularity this window ranged between 90 and 130 ms, whereas for the pattern regularity, the range was between 130 and 170 ms. A repeated measures analysis of variance (ANOVA) with the factors Frequency (650 and 800 Hz) and Stimulus Type (Standard, Deviant) was calculated on the mean amplitudes extracted from the MMN time window.

For the MLR range, data were filtered using a band-pass FIR filter from 15 to 250 Hz. Epochs of 150 ms were used, including a −50 ms baseline. Trials with amplitudes larger than ±80 µV were rejected from further analysis. Epochs were averaged separately for standard and deviant tones. Standards of the pattern regularity were the tones presented before the repetition deviants, whereas the standards of the reverse block were used for the simple regularity. Grand average peak latencies for repetition and ITD deviants were extracted from each MLR component (P0, Na, Pa and Nb), and they were the same for both deviant types. Individual mean amplitudes were extracted from a 4 ms window centered on the grand average peak latency. Therefore, mean amplitudes were obtained for latencies between 11–15 ms (P0), 21–25 ms (Na), 30–34 ms (Pb) and 41–45 ms (Nb). Similar to the MMN analysis, a repeated measures ANOVA with the factors Frequency (650 and 800 Hz) and Stimulus Type (Standard, Deviant) was calculated for each MLR component.

Additionally, when significant differences between standards and deviants were observed at Fz for the simple regularity, we performed a separate analysis to explore the laterality effects on both ranges (MLR and MMN). Mean amplitudes were extracted at electrodes F3 (left hemisphere) and F4 (right hemisphere) and a repeated measures ANOVA with the factors Stimulus Type (Standard or Deviant) and Hemisphere (F3 and F4) was conducted for each frequency.

EEG data analysis was performed with EEGLAB [33]. For the statistical analysis, Bonferroni corrections for multiple comparisons were performed when comparing several MLR components, and in those cases that *post hoc* pairwise comparisons were required.

## Results

**Figure 2:**
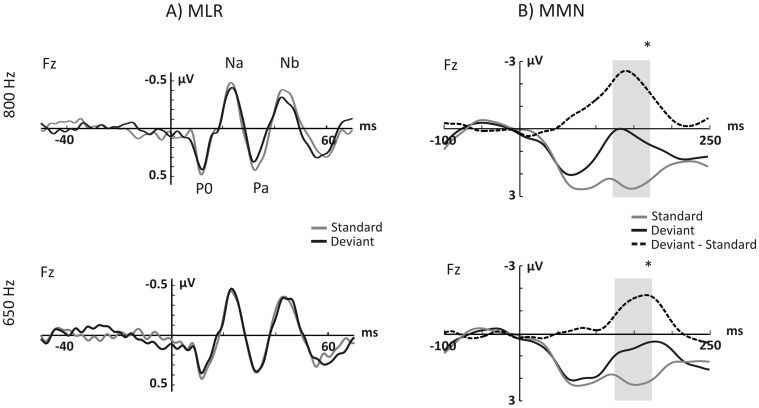
AEPs for the pattern regularity. Grand average evoked potentials (N = 24) elicited by standard tones (grey lines), and deviant tones (black lines) at the Fz electrode. The upper and lower rows show the responses to the 800 and 650 Hz tones, respectively. A) Waveforms in the MLR range. B) Waveforms in the MMN range. Dashed lines show the MMN elicited by the repetition deviants. The grey bars denote the windows of measurement. [*p<0.001].

**Figure 3:**
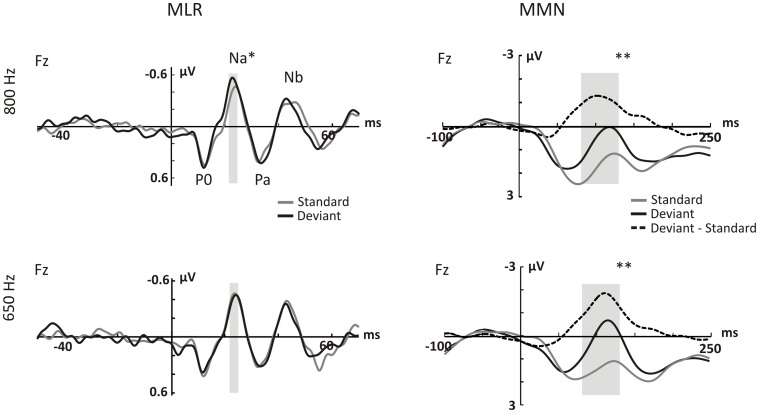
AEPs for the simple regularity (ITD change). Grand average evoked potentials (N = 24) elicited by standard tones (grey lines), and deviant tones (black lines) at the Fz electrode. Note that standard tones for the simple regularity correspond to the standard tones in the reverse block, which had the same physical characteristics as the ITD deviants in the tone-alternation sequence. The upper and lower rows show the responses to the 800 and 650 Hz tones, respectively. A) Waveforms in the MLR range. B) Waveforms in the MMN range. Dashed lines show the MMN elicited by the ITD deviants. The grey bars denote the windows of measurement in A) and B). [*p<0.05; **p<0.001].

For each type of regularity violation (pattern and simple), MMN and MLR results are reported (see tables 1 and 2 for mean MMN and MLR component amplitudes). The corresponding MMN and MLR waveforms are shown in Figures 2 and 3.

**Table 1:** Pattern regularity: mean amplitudes of the P0, Na, Pa and Nb components of the MLR and the MMN range.

Pattern					
Mean amplitude (std error)					
	P0	Na	Pa	Nb	MMN
*650 Hz tone*					
**Std**	0.38 (0.06)	−0.39 (0.06)	0.31 (0.06)	−0.36 (0.06)	2.13 (0.18)
**Dev**	0.32 (0.06)	−0.39 (0.05)	0.30 (0.07)	−0.33 (0.06)	0.60 (0.31)*
*800 Hz tone*					
**Std**	0.40 (0.05)	−0.41 (0.05)	0.35 (0.07)	−0.35 (0.08)	2.59 (0.28)
**Dev**	0.36 (0.04)	−0.39 (0.05)	0.28 (0.07)	−0.33 (0.07)	0.28 (0.33)*

Mean amplitudes (in µV) and standard errors (in parentheses) elicited by standard (std) and deviant (dev) tones for each MLR component and the MMN range at the Fz electrode. Asterisks indicate significant differences between standard and deviant responses [*p<0.001].

**Table 2:** Simple regularity (ITD change): mean amplitudes of the P0, Na, Pa and Nb components of the MLR and the MMN range.

Simple (ITD change)					
Mean amplitude (std error)					
	P0	Na	Pa	Nb	MMN
*650 Hz tone*					
**Std**	0.36 (0.04)	−0.36 (0.06)	0.25 (0.07)	−0.32 (0.07)	1.32 (0.21)
**Dev**	0.32 (0.04)	−0.33 (0.07)	0.24 (0.07)	−0.30 (0.08)	−0.33 (0.32)**
*800 Hz tone*					
**Std**	0.39 (0.05)	−0.35 (0.05)	0.32 (0.07)	−0.25 (0.09)	1.55 (0.19)
**Dev**	0.39 (0.05)	−0.49 (0.06)*	0.30 (0.06)	−0.28 (0.06)	0.31 (0.28)**

Mean amplitudes (in µV) and standard errors (in parentheses) elicited by standard (std) and deviant (dev) tones for the Na component of the MLR and the MMN range at the Fz electrode. Note that standard tones for the simple regularity correspond to the standard tones in the reverse block, which had the same physical characteristics as the deviants in the tone-alternation sequence. Asterisks indicate significant differences between standard and deviant responses [*p<0.05; **p<0.001].

### Pattern Regularity

The repetition of either one of the two tones elicited a MMN that peaked at about 150 ms after stimulus onset. The repeated measures ANOVA revealed no main effect of Frequency (*F*(1,23) = 0.453, *p = *0.508), but there was a main effect of Stimulus Type (*F*(1,23) = 97.678, *p*<0.001). Additionally, a Frequency by Stimulus interaction was observed (*F*(1,23) = 6.005, *p = *0.022). The latter resulted from the amplitude differences between MMNs elicited by low (650 Hz) and high (800 Hz) frequencies (*post hoc* pairwise comparisons: *t*(23) = 6.166, corrected *p*<0.001 for 650 Hz tones; *t*(23) = 9.263, corrected *p*<0.001 for 800 Hz tones), with larger differences between standard and deviant responses observed in the high frequency (650 Hz: mean = 1.54 µV, SEM = 1.23; 800 Hz: mean = 2.31 µV, SEM = 1.22).

In the MLR range, no main effect of Frequency was observed on any of the MLR components amplitudes (*F*’s<0.302, corrected *p*’s >0.999), nor was there a Stimulus Type effect (*F*’s<0.649, corrected *p*’s >0.428, respectively). Similarly, there was no Frequency by Stimulus Type interaction on any of the MLR components (*F*’s<0.459, *corrected p*’s >0.999).

### Simple Regularity

MMN elicited by ITD deviants peaked earlier than MMN generated by repetition deviants, at about 110 ms after stimulus onset. The repeated measures ANOVA revealed a main effect of Frequency (*F*(1,23) = 16.435, *p*<0.001) and Stimulus Type (*F*(1,23) = 43.970, *p*<0.001). Moreover, a trend towards a Frequency by Stimulus Type interaction was observed (*F*(1,23) = 4.202, *p = *0.052).

In the MLR range there was no main effect of Frequency (*F*’s<2.593, *corrected p*’s >0.242), nor was there any Stimulus Type effect on any of the MLR components amplitudes (F’s<0.209, *corrected p*’s >0.442). Similarly, no Frequency by Stimulus Type interaction was observed at P0 (*F*(23) = 0.306, *corrected* p>0.999), Pa (*F*(1,23) = 0.018, *corrected p = *0.895) and Nb (*F*(1,23) = 0.491, *corrected p = *0.491). However, a Frequency by Stimulus Type interaction was observed at Na (*F*(1,23) = 10.062, *corrected p = *0.016). *Post hoc* pairwise comparisons showed that deviant responses were larger than the standard responses for the 800 Hz tones (*t*(23) = 2.611, *corrected p = *0.032), but not for the 650 Hz tones (*t*(23) = −0.665, *corrected p*>0.999).

**Figure 4:**
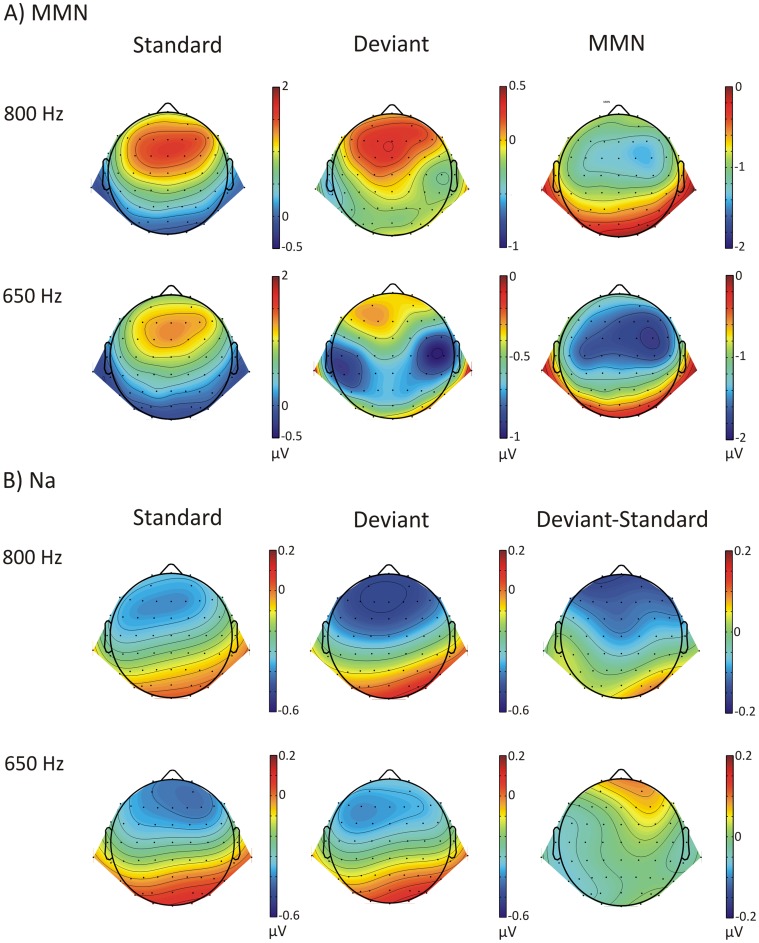
Scalp topographies for the simple regularity (ITD change). A) Long-latency responses (90–130 ms time window) to the standard tones of the reverse block, the deviant tones and the MMN. B) MLR responses at Na (21–25 ms time window) to the standard tones of the reverse block, the deviant tones and their difference (deviant-standard).

The general assessment of laterality effects for ITD deviants in the MMN range for the 650 Hz tone showed a main effect of Stimulus Type (*F*(1,23) = 38.632, *p*<0.001), but there was no main effect of Hemisphere (*F*(1,23) = 38.632, *p = *0.428). However, an interaction between Stimulus Type and Hemisphere was observed (*F*(1,23) = 7.951, *corrected p = *0.010). *Post hoc* pairwise comparisons revealed significant differences between deviant and standard tones on the left hemisphere (*t*(23) = 5.320, *corrected p*<0.001) as well as on the right hemisphere (*t*(23) = 6.975, *corrected p*<0.001), with larger MMN amplitudes elicited on the right hemisphere (F4 mean = 1.74 µV, SEM = 0.25) than the left hemisphere (F3 mean = 1.46 µV, SEM = 0.27). For the 800 Hz frequency, a main effect of Stimulus Type was observed (*F*(1,23) = 40.389, *p*<0.001), but there was no main effect of Hemisphere (*F*(1,23) = 0.701, *p = *0.411). However, there was a Stimulus Type by Hemisphere interaction (*F*(1,23) = 4.667, *p = *0.041). MMN amplitudes were significant on both left (*t*(23) = 5.550, *corrected p*<0.001) and right hemispheres (*t*(23) = 7.115, *corrected p*<0.001), with larger amplitudes observed over the right hemisphere (F4 mean: 1.30, SEM = 0.18; F3 mean = 1.16, SEM = 0.18). In the MLR range, the laterality assessment of the Na component for the 800 Hz frequency revealed a main effect of Stimulus Type (800 Hz: *F*(1,23 = 4.757, *p = *0.040); but there was no main effect of Hemisphere (*F*(1,23) = 0.514, *p = *0.481). Similarly, no interaction between Stimulus Type and Hemisphere was observed (*F*(1,23) = 0.626, *p = *0.437). Scalp topographies for MMN and the Na component of the MLR are shown in Figure 4.

## Discussion

In the present study, two levels of auditory deviance detection were examined for two different types of auditory regularities. First, a pattern regularity was defined by the alternation of two tones, which was violated by an infrequent repetition of either one of the tones. In this case, the system requires encoding the pattern regularity of the sequence, which is determined by the relationship between successive stimuli [34]. Second, a variation of the perceived location of the tones was induced by introducing an ITD change in the deviants. Here, the regularity was represented by a feature trace that was constant along the sequence. As we expected, violations to both types of regularities elicited MMN. In the MLR range, no evidence for early auditory change detection was observed for violations of the pattern regularity, whereas for the simple regularity a modulation of the Na component was found when the ITD deviants occurred in the 800 Hz tones. Therefore, the differences between both time ranges support the view of a hierarchical organization of auditory deviance detection processing in the auditory system.

To our knowledge, this is the first study to probe the processing of violations of a pattern regularity in a latency range preceding the MMN. Previous oddball studies showed evidence for early auditory deviance detection in the brainstem frequency-following response to consonant-vowel deviants [35], and in the MLR components to changes in frequency [22], [23], intensity [24], location [19]–[20] and band-pass filtered noise bursts [21]. However, by oddball paradigms alone it is not possible to determine whether the deviance-related modulations of the responses are feature-specific. By introducing the simple (ITD) regularity within the tone-alternating sequence we were able to observe whether deviance detection effects due to the variations of one of the stimulus characteristics existed in the MLR components.

In the MLR range, the effects we observed in the Na component when the ITD deviants occurred resemble the findings of previous studies that examined location changes in oddball paradigms. Specifically, Sonnadara *et al.* (2006) reported an enhancement of the Na component by using band-pass filtered noise bursts whose perceived location was varied by using head-related transfer functions [20]. Grimm *et al.* (2012) confirmed that the Na enhancement was indeed the result of a genuine deviance detection process, by presenting click stimuli in free field, with an additional condition to control for refractoriness confounds [19]. By means of intracerebral recordings, the Na component sources have been localized at the posteromedial part of Heschl’s gyrus, corresponding to A1 [36]. Our MLR findings thus suggest that deviance detection to a simple feature occurred at low hierarchical regions of the auditory cortex.

In terms of the long latency range, MMN responses were observed for both tone repetitions and ITD changes, which were consistent with previous findings [26]–[29], [37]–[40]. Moreover, MMN generated by the single feature ITD change peaked earlier than the MMN generated by the pattern violations. This latency difference between simple and pattern regularity violations suggests that, at the later AEP range, these two types of regularities are also processed differently. A similar latency effect was previously reported between an oddball and a tone-alternation paradigm, although the authors failed to observe a significant repetition MMN [41]. Our results thus confirm that violations to both simple and pattern regularities could be processed higher in the hierarchy of the auditory deviance detection system, with the simple ITD change activating earlier deviance detection mechanisms.

Taken together, our MLR and MMN findings support the notion that auditory deviance detection might occur in a hierarchical manner [42]. In an early stage of the auditory hierarchy our results confirm that deviance detection mechanisms occur for single-feature deviants. The repetition deviants might not be detected until later in the hierarchy, probably due to the need of higher-order mechanisms to encode the tone-alternating regularity. In a later stage of the auditory hierarchy, MMN elicitation might not only reflect deviance detection mechanisms, but an additional update of the underlying acoustic model [43]–[44], that under certain conditions (e.g in case of simple regularities) might pass its predictions to lower stages of the auditory pathway.

Even though we were able to observe enhanced responses to the ITD deviants in the MLR range, the enhancement was only present in one of the two tones of our tone-alternating paradigm. These differences might be due to the use of 700 µs ITDs for both tones (left ear leading). By introducing the same ITD in both frequencies, we induced an interaural phase delay (IPD) in the 800 Hz tones that could be interpreted as right ear leading, since our ITD delay was larger than half the period of this frequency. Therefore, for the 800 Hz tones, there was an ambiguity between the onset disparity (left ear leading) and the IPD (right ear leading). This ambiguity could have provided an additional sensory cue that was not present in the 650 Hz tones. The lack of deviance-related effects in the 650 Hz tones in the MLR range, are in contrast with our findings in the MMN range and with previous MMN studies that showed MMN elicitation when introducing large IPDs [e.g. 37], and even reported enhanced MMN amplitudes when the sounds were perceived as more far-lateralized [38]. As no deviance-related effects were observed for the 650 Hz when applying a large IPD it remains to be addressed what the effects of smaller IPDs would be in the MLRs.

Regarding the lack of evidence to early change detection mechanisms when the tone-alternation regularity was violated, we cannot rule out the possibility that any deviance-related enhancement may have been outweighed by an amplitude suppression due to repetition. In a previous study, Müller et al. (2001) showed that, in a paired-click paradigm, significant amplitude suppression was found at several components of the MLR, starting as early as at the Na component [45]. However, it has been suggested that repetition suppression is more enhanced when repetitions are expected (e.g. when presenting pairs of tones), than when they are unexpected, suggesting the role of top-down expectations [46]. In our paradigm repetitions violating the pattern regularity occurred randomly and with a low probability. We would expect that this unpredictability diminished any repetition suppression, but we cannot determine to what extent a potential suppression effect might still have outweighed deviance detection effects. Follow-up studies are needed to further examine the distinct contributions of possible repetition suppression amongst expected versus unexpected repetitions in this paradigm.

One might speculate that the differences observed in our two latency ranges might be explained by the existence of local regularity extraction mechanisms. Specifically, complex auditory regularities might not be encoded at ranges earlier than MMN, whereas auditory deviance detection of simple features such as location cues, intensity or frequency may occur at lower hierarchical levels. Our simple feature change findings, suggest that ITD per se was encoded as a separate feature in the lower and higher levels of the hierarchy. The fact that our MLR findings for ITD deviants were not generalized to both frequencies -while our MMN findings were- might be explained by the characteristics of auditory space encoding in different cortical levels, possibly becoming more accurate in higher cortical areas [47]. A possible interpretation for the missing repetition-deviance effect in the MLR latency range is that the pattern regularities are yet to be extracted and represented at the initial levels of stimulus processing. In this regard, the pattern regularity of alternating tones might be encoded as an equiprobable representation of the two frequencies, eliciting similar responses for both standard and deviant tones. This suggests that a complex mechanism may be required, probably not taking place until later stages as shown by the MMN elicitation. However, as mentioned above, we can only speculate whether it is indeed the process of regularity extraction that is accomplished for simple feature repetition rules at earlier levels in the auditory hierarchy. Alternatively, one could hypothesize that regularity detection occurs always at higher stages, yet in the case of simple regularities predictive signals will be passed down to lower levels of the hierarchy that allow the detection of deviants at an earlier stage.

Furthermore, we studied the hemispheric lateralization of the observed deviance-related effects in both early and late latency ranges. Our results revealed contralateral dominance to the perceived change of sound location in the MMN range. These findings are in accordance with previous MMN studies that presented the stimuli with ITD variations via headphones [39], [48]. Conversely, for the Na component we did not find a contralateral (right) dominance to the stimuli perceived as coming from the left. These findings are in contrast with the laterality effects observed by Sonnadara et al. (2006) for stimuli presented at −30° [20], and by Grimm et al. (2012) for the Na difference response between standard and deviant stimuli [19]. Such discrepancies could be related to our use of large ITDs resulting in somewhat ambiguous location cues for the 800 Hz ITD deviants, or to the fact that in both previous studies more location cues could be integrated. Nevertheless, the different patterns in scalp topographies for MMN and Na may suggest the existence of different neural generators.

To summarize, we were able to show evidence for auditory deviance detection at two different levels of the auditory hierarchy. In the MLR range, early deviance detection occurred at the Na component for simple feature (ITD) deviants, but not for pattern deviants. MMN elicitation to both types of deviants showed that at a higher level of the hierarchy, simple and pattern regularity violations were detected, and probably additional mechanisms such as an update of the acoustic representations took place. The differences between a frequency-specific ITD deviance detection in the MLR range, as opposed to the long-latency range, suggest that a more accurate deviance detection mechanism may not occur until later stages, or it may require additional information from other types of cues, such as ILDs or spectral cues. Nevertheless, our findings reflect the existence two levels of deviance extraction mechanisms, operating at different levels of complexity. Further studies will be needed in order to elucidate how these two levels of auditory change detection interact with each other.
